# A case of spontaneous resolution of a scrotal ventriculoperitoneal shunt migration

**DOI:** 10.1016/j.radcr.2022.07.039

**Published:** 2022-07-29

**Authors:** Anas Alkhudari, Maad Galal, Zainab Wagley, Belal Nedal Sabbah, Abdelrafour Houdane, Aljohara Aljabr

**Affiliations:** a College of Medicine, Alfaisal University, Al Takhassousi, Al Zahrawi Street, Riyadh 11533, Kingdom of Saudi Arabia; b Department of Radiology, King Fahad Medical City, Riyadh, Kingdom of Saudi Arabia

**Keywords:** Scrotal ventriculoperitoneal shunt, Spontaneous resolution, Infant hydrocephalus, Neurosurgery

## Abstract

Ventriculoperitoneal (VP) shunt catheter migration remains a rare but documented complication seen in one in 1000 patients who receive a VP shunt. Migration of the VP shunt into the scrotum is even more uncommon and requires surgical treatment. We report a unique case of a 6-month-old preterm male who developed right scrotal migration of his VP shunt. However, the tip of the VP shunt spontaneously reduced to its normal position, and repeated imaging months later showed no recurrence.

## Introduction

In the treatment of infant hydrocephalus, ventriculoperitoneal (VP) shunts are considered standard of care. They aim to redirect cerebrospinal fluid from the ventricles toward other compartments to decrease intracerebral pressure. In the United States, VP shunt placement remains a standard neurosurgical procedure accounting for more than 30,000 hospital admissions annually [1]. Despite the drastic improvement in morbidity and mortality rates due to hydrocephalus in pediatrics, various complications are associated with VP shunting [1]. Complications include infection, bleeding, and CSF leaks, but shunt dysfunction is considered the most common neurosurgical complication seen [2]. Distal catheter migration is an uncommon complication in only one in 1000 patients [3]. The gastrointestinal tract, bladder, abdominal wall, vagina, scrotum, and mediastinum are all locations of migration that has been reported in the literature [4]. Scrotal migration remains a very rare complication, with recent systemic reviews showing only 48 reported cases in the literature [5,6]. Treatment was surgical in all but one case reported in 1983 [5]. We present a unique case of a 6-month-old male with right scrotal migration confirmed by medical imaging that spontaneously resolved without medical or surgical intervention.

## Case presentation

**Fig. 1:**
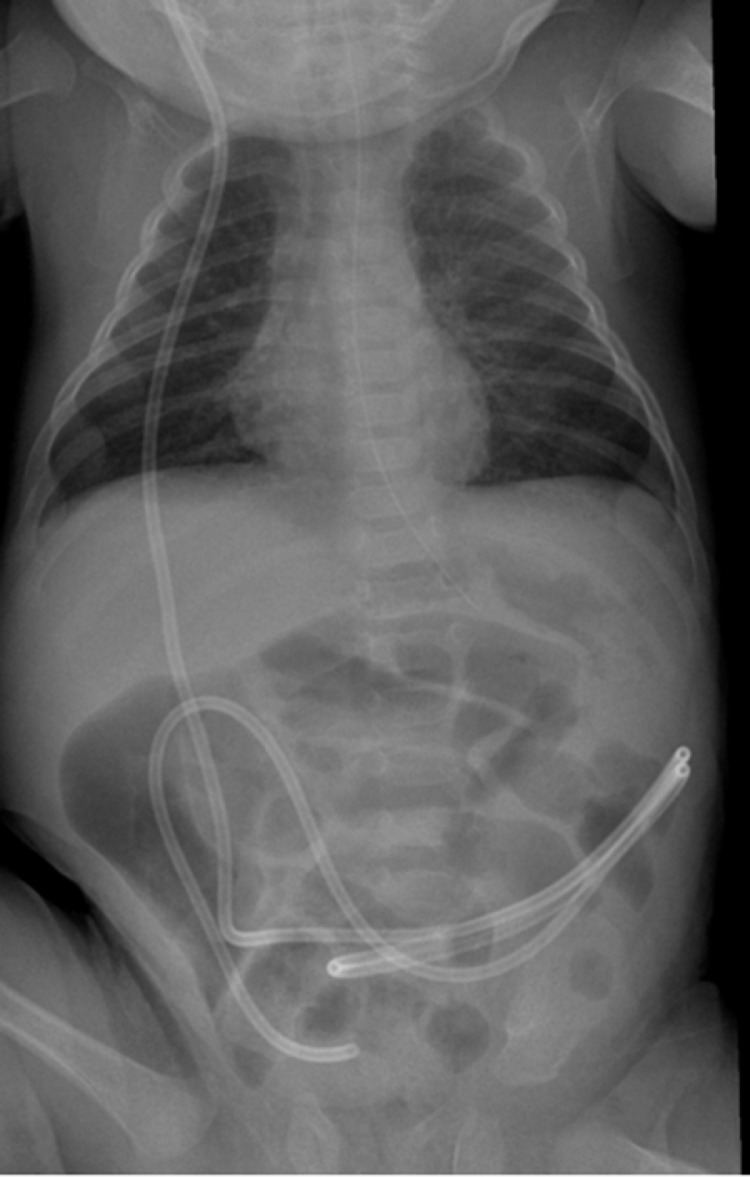
The distal tip of the VP shunt is projected over the right lower quadrant.

A 6-month-old preterm (28 weeks) 6-month-old male (corrected age 3 months) was referred to our hospital with complaints of projectile, non-bloody, and non-bilious vomiting. This was ongoing for the previous 15 days and happened after each feed. The vomiting was also associated with constipation for 6 days, followed by the passage of a large watery stool upon being triaged. The patient had an extensive medical and surgical history, significant for hydrocephalus secondary to intraventricular hemorrhage (Grade III), periventricular leukomalacia, epilepsy, hemangiomas, epispadias, and bilateral reducible hernias. The patient's surgical history revealed a surgically placed VP shunt on the right side through a frontal approach to treat his hydrocephalus. The images after insertion of the VP shunt showed proper positioning (Fig. 1), and a repeat CT brain and shunt series confirmed an intact shunt with no signs of radiological failure. A head ultrasound later showed the tip in the right lateral ventricle and improvement of ventricular dilation.

On physical examination, the patient appeared to be well without any signs of distress. The anterior fontanel was flat, and both pupils were equal and reactive. The abdomen was soft and lax with bilateral inguinal hernias that were reducible and not strangulated. Both testes were palpated in the scrotum. The patient had a right inguinoscrotal sac swelling with the tip of the catheter appreciated upon palpation of the sac. Laboratory examinations suggest mild dehydration without any signs of an underlying infectious process.

**Fig. 2:**
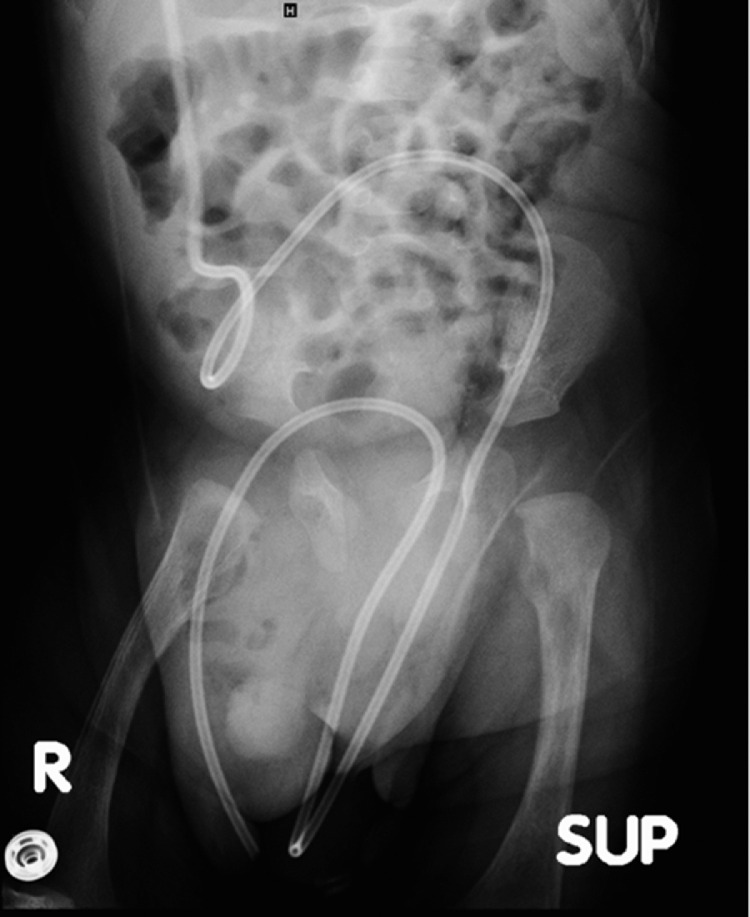
Follow-up abdomen radiograph showing distal tip of VP shunt projects over right scrotal sac.

**Fig. 3:**
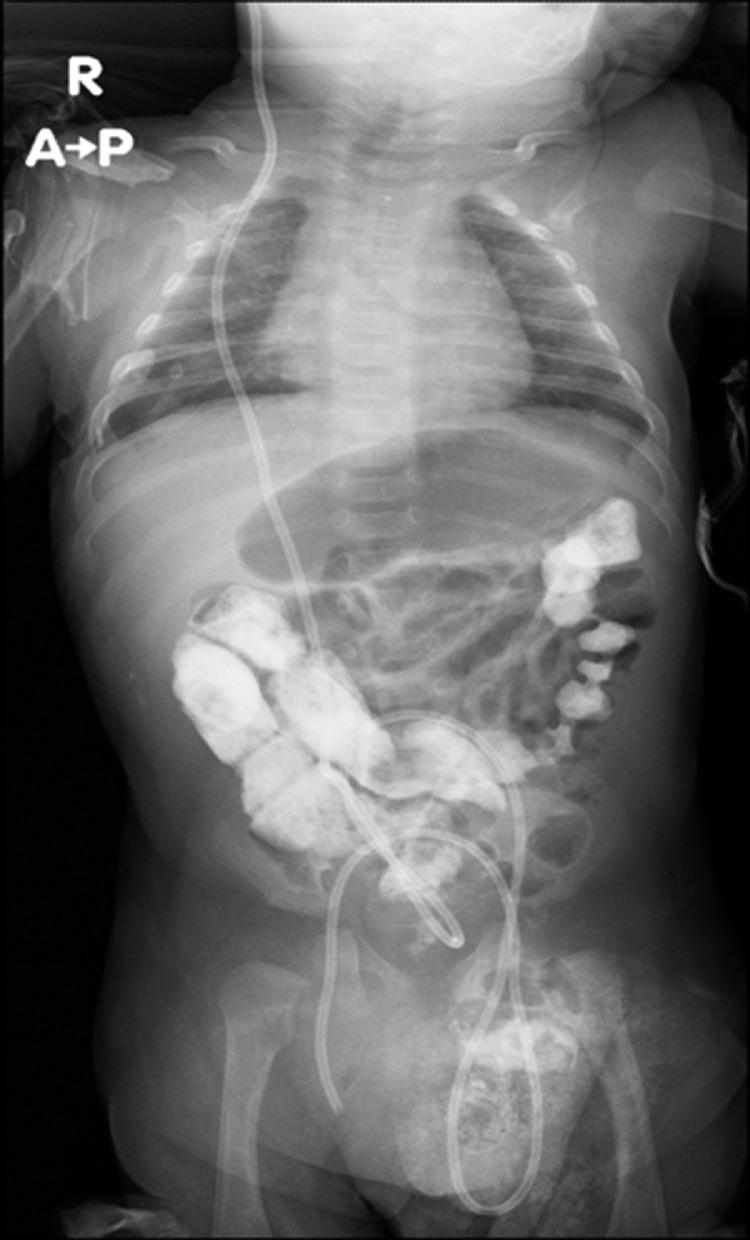
Follow-up abdominal radiograph, unchanged.

On the basis of the clinical picture, an abdominal ultrasound was ordered to rule out hypertrophic pyloric stenosis, which returned negative. Upon further imaging by abdominal radiograph, the distal catheter tip of the VP shunt was seen projecting over the right scrotal sac, confirming the migration of the shunt migration (Fig. 2, Fig. 3).

**Fig. 4:**
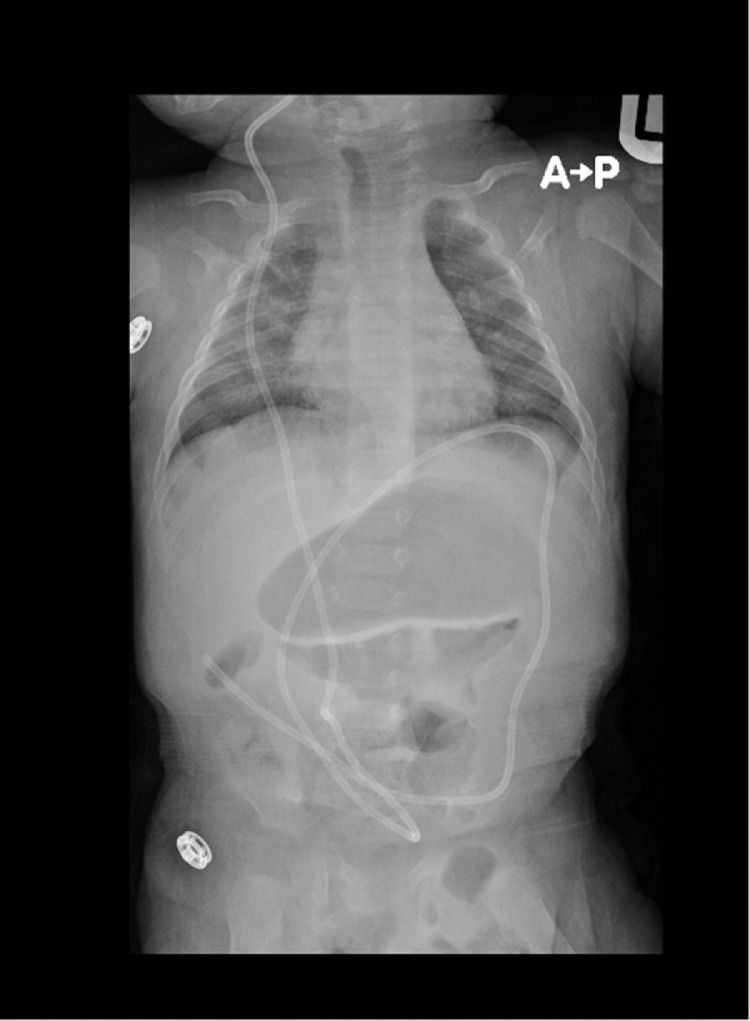
Abdominal radiograph postreduction.

After admission, the patient started to show rapid and significant improvement without significant intervention. On further imaging, the positioning of the VP shunt improved with a sufficiently reduced tip (Fig. 4). The patient was discharged without further complications. One month later, the patient presented to the emergency room with an episode of vomiting. Repeated imaging showed no movement of the VP shunt with no recurrence of migration, highlighting the spontaneous reduction of the distal catheter tip.

## Discussion

There are several hypotheses in the literature regarding the etiology of VP shunt migration, but the exact cause remains undetermined. These include the firmness of the catheter used, focal wound dehiscence, poor host immunity, improper surgical technique, or ischemic necrosis of the dermis as potential factors [7]. Scrotal migration remains uncommon, with around 50 cases reported in the literature so far [5]. The median age at presentation was 13.5 months, and most migrations occurred on the right side. All patients presented scrotal swelling, with 81% of patients presenting only a scrotal mass.

Scrotal migrations are more common in children, and this is commonly thought to be due to an unobliterated processus vaginalis allowing the catheter tip to enter the scrotal sac [8]. The processus vaginalis usually remains patent for the first year of life, explaining the incidence of inguinal problems like hydrocele and inguinal hernias [3]. Increased abdominal pressure is believed to be a contributing factor, and children are more prone to exceed the maximum CSF absorption rate of the VP shunt, increasing intra-abdominal pressure and volume [9].

We hypothesize that preterm patients have an increased risk of migration to the scrotum due to having a smaller abdominal cavity and a higher incidence of non-closure of the vaginal process. Previous studies [5] have shown that preterm patients are at an increased risk of inguinal hernia secondary to a similar process. As most patients remain asymptomatic and present only after an exacerbation with co-infections, the importance of early diagnosis should not be neglected. This is crucial to avoid subsequent complications such as acute hydrocephalus, testicular torsion, and peritoneal perforation [7,10].

Spontaneous resolution of scrotal VP shunt migration is incredibly rare, having only ever been reported once before in the literature back in 1983. This highlights the possibility that the rate of scrotal shunt migration could be higher than reported in the literature if spontaneous resolution is a possible outcome. This could be explored once more case reports emerge in the literature.

For nearly all scrotal VP shunt migration cases, definitive treatment is surgical with manual repositioning followed by hernia repair. A treatment algorithm proposed by Hauser et al. is recommended based on a systemic review of all existing cases [5]. A trial of outpatient manual repositions, and reduction is tried first, with elective hernia repair if successful. Failure of manual reduction warrants surgical repositioning and hernia repair through an inguinal incision (with room for an additional scrotal incision if needed).

## Conclusion

We recommend that all infants, especially preterm ones, receive frequent scrotal exams during their follow-up appointments at the neurosurgery clinic. VP shunt migration should remain an essential differential of scrotal masses in that patient population, and early surgical intervention based on the Hauser algorithm should be considered. We also want to raise awareness to radiologists about the malposition of the VP shunt catheter and the rare ability for it to reduce and retract into appropriate positioning spontaneously. We believe that these recommendations will help improve quality of care management in pediatric hydrocephalus patients.

## Authors’ contributions

A.A., M.G., Z.W., B.N.S., A.H. drafted the manuscript B.N.S. and A.H. contributed to reviewing and finalizing the manuscript. Al.A provided the imaging findings and their interpretation for the case presentation section. All authors reviewed the manuscript for intellectual content and approved the submission.

## Ethics approval

Patient anonymity is maintained throughout this manuscript, and consent was obtained for publication from the patient's parents.

## Patient consent

Written informed consent was obtained from the patient's family for publication of this case report and accompanying images. Patient anonymity is maintained throughout this manuscript.
